# Pollution of Streams

**Published:** 1895-03

**Authors:** 


POLLUTION OF STREAMS.—It is gratifying to observe a gradual awakening of the public to realization that prevention is not only "better than cure," but also much cheaper. We see that in several States bills have been introduced looking to a preservation of the general health, and recently in Congress there has been a bill introduced, which, if it become a law, will be far reaching and comprehensive for good. The oyster epidemic, of which we made mention last month, served as a forcible object lesson, and occurring as it did, up there in God's country, New England, it moved the people to action.

The pollution of streams is one of the most fruitful sources of sickness, and especially, of typhoid fever. It can only be prevented by the closest watch, and the enforcement of the most rigid measures; and while we are in for "State rights," and believe it is the duty of each State to look after the health of its own people, yet if the State will not do it the United States government should do it; it is a matter of such importance that we should not stickle on form, or stand on the manner of doing it, so it is done, and well done. In the matter of preventing the pollution of running streams it would be impossible we apprehend, to secure co-operation of all States interested in, or through which runs any particular river or stream, and no amount of watchful care on the part of one State would insure safety to those States through which the stream runs; hence, it becomes imperative upon the United States government to take the matter in hand.

Congressman Bartholdt, of Missouri, has formulated a bill to this effect, and it has been introduced into Congress. We give below the provisions of the bill. It provides for a commission, to be appointed by the President, and is in substance as follows:

"That a commission shall be appointed to investigate fully the subject of the pollution of rivers and other natural sources of water supply where the sanitary condition of the people of more than one State is affected or threatened to be affected by such pollution, this commission to consist of three members, to be appointed by the president, by and with the consent of the senate, whose compensation during the time when actually engaged in the performance of their duties under this act, shall be $10 per diem each and reasonable expenses.

"Section 2. The commission shall meet in Washington, D. C., within 30 days after the passage of this act, to consider the methods to be adopted in the investigation, and it shall have authority and be empowered to make use of the services of chemical, bacteriological and sanitary experts, and of such persons as it may judge most competent by reason of their special knowledge and experience to afford it correct information on the subject of its inquiry, as well as in formulating its methods and in carrying them into effect. It shall meet thereafter from time to time at such places as it may consider best suited for the furtherance of its inquiry.

"Section 3. The commission shall report to Congress at its next session the progress made in the investigation undertaken under this act, and shall submit such suggestions as may seem desirable with the view of remedying any insanitary conditions that have been developed by its work."

Dr. George Homan, Health Commissioner, discussed the question with Major Charles Smart, Surgeon of the United States Army; an expert chemist and analyser of water, who has done a great deal of work for the National Board of Health, at the last annual meeting of the American Public Health Association at Montreal. At Dr. Homan's suggestion last month Dr. Smart drafted this bill, and in his letter submitting it to Dr. Homan, said that the bill met with the approval of Dr. Sternberg, Surgeon General of the United States Army. Before it was forwarded to Congressman Bartholdt the bill was submitted to and approved by Mayor Walbridge.—Exchange.

